# Critical Role of the Presynaptic Protein CAST in Maintaining the Photoreceptor Ribbon Synapse Triad

**DOI:** 10.3390/ijms24087251

**Published:** 2023-04-14

**Authors:** Akari Hagiwara, Ayako Mizutani, Saki Kawamura, Manabu Abe, Yamato Hida, Kenji Sakimura, Toshihisa Ohtsuka

**Affiliations:** 1 Department of Applied Biological Science, Faculty of Science and Technology, Tokyo University of Science, Chiba 278-8510, Japan; 2 Department of Biochemistry, Faculty of Medicine, University of Yamanashi, Yamanashi 409-3898, Japan; 3 Department of Animal Model Development, Brain Research Institute, Niigata University, Niigata 951-8585, Japan

**Keywords:** ribbon synapse, retina, CAST, ELKS, photoreceptor, horizontal cell

## Abstract

The cytomatrix at the active zone-associated structural protein (CAST) and its homologue, named ELKS, being rich in glutamate (E), leucine (L), lysine (K), and serine (S), belong to a family of proteins that organize presynaptic active zones at nerve terminals. These proteins interact with other active zone proteins, including RIMs, Munc13s, Bassoon, and the β subunit of Ca^2+^ channels, and have various roles in neurotransmitter release. A previous study showed that depletion of CAST/ELKS in the retina causes morphological changes and functional impairment of this structure. In this study, we investigated the roles of CAST and ELKS in ectopic synapse localization. We found that the involvement of these proteins in ribbon synapse distribution is complex. Unexpectedly, CAST and ELKS, in photoreceptors or in horizontal cells, did not play a major role in ribbon synapse ectopic localization. However, depletion of CAST and ELKS in the mature retina resulted in degeneration of the photoreceptors. These findings suggest that CAST and ELKS play critical roles in maintaining neural signal transduction in the retina, but the regulation of photoreceptor triad synapse distribution is not solely dependent on their actions within photoreceptors and horizontal cells.

## 1. Introduction

Retinal processing neurons, including light-sensitive photoreceptors, are organized in laminar structures that form numerous parallel microcircuits. Signals evoked by light stimulation of photoreceptors are conveyed by bipolar cells to ganglion cells, which in turn, transmit the signal to the central nervous system (CNS) via the optic tract. This neural information is processed by horizontal and amacrine cells *en route*. During early development, neurons migrate toward their target positions using multiple signaling pathways [1], and dysregulated cues cause the ectopic distribution of neurons, resulting in various deficits [2]. Therefore, morphologically accurate neural and synaptic localization is essential for functional neural networks.

In the mouse retina, cones are generated prenatally as early as embryonic day (E) 11. Rods are generated from E12 to postnatal day (P) 10. During that time, the eyes of the newborn pup remain closed for almost 2 weeks. As photoreceptors mature, the opsin levels increase substantially, and the outer segments elaborate toward the retinal pigment epithelium (RPE) [3]. The neural laminar structures are consolidated by the formation of synapses. The photoreceptor ribbon synapse has a unique triad structure with bipolar and horizontal tips. The triad is localized in the outer plexiform layer (OPL) between the photoreceptor layer (outer nuclear layer, ONL) and the bipolar cell layer (inner nuclear layer, INL). In some cases of photoreceptor degeneration, such as those caused by conditional knockout (cKO) of the retinitis pigmentosa gene, Pomt1 [4,5], and in the rhodopsin and cyclic nucleotide-gated A3 (CNGA3) double knockout (KO) [6], the photoreceptor cell body initially localizes to the proper layer, but the ribbon synapse ectopically localizes to the ONL.

We previously reported that ribbon synapse ectopic localization in the ONL leads to the aberrant outgrowth of bipolar and horizontal processes in the retinas of CAST KO and CAST/ELKS double KO animals [7,8]. The ectopic ribbon synapse was significantly increased in the CAST/ELKS double KO mouse, but photoreceptor degeneration was not detected in the 10- or 40-week retina. The ectopic ribbon synapse is demonstrated well in the nob2 (no b-wave 2) mouse, a naturally occurring mutant caused by a null mutation in Cacna1f [9,10]. Mutations in Cacna1f, which encodes the α1F subunit of Cav1.4, cause the X-linked heritable disease incomplete congenital stationary night blindness (CSNB2) in humans [11]. A characteristic of CSNB2 is a normal a-wave, but a severely reduced b-wave in the dark-adapted electroretinogram, indicating intact phototransduction in photoreceptors, but disrupted synaptic transmission to bipolar cells [9]. Furthermore, age-associated remodeling, which is regulated by the serine/threonine kinase LKB1 and AMPK, is characterized by increased ectopic ribbon synapses [12,13].

Like the nob2 mouse, the CAST KO and the CAST/ELKS double KO exhibit a normal a-wave, but transmission to bipolar cells, indicated by the b-wave, is reduced by ~50% in the CAST KO, and by ~80% in the CAST/ELKS double KO [7,8]. Furthermore, ectopic localization of ribbon synapses to the ONL and a reduction in the b-wave is also found with KO of the active zone component protein, Bassoon [14]. Intriguingly, the terminals are ultrastructurally characterized by the presence of “floating” ribbons in the Bassoon KO and in the nob2 mouse, while the ribbon is intact at bipolar cell postsynaptic sites in the CAST KO and the CAST/ELKS double KO [8,14,15]. Given the abundance of the major protein component of the ribbon synapse, RIBEYE, it is difficult to evaluate ectopic distribution, particular when the ribbon synapse is absent [16]. However, the terminal and postsynaptic components seem to penetrate the ONL. These observations suggest that the disruption of photoreceptor transmission may result in morphological changes, including mislocalization of ribbon synapses in the OPL. Thus, the ectopic ribbon synapse, a morphological feature pathognomonic of diminished vision, varies in distribution pattern, depending on the cause and degree of blindness.

Horizontal cells play a complex role in visual information relay, forming an electrically-coupled network that provides feedback signals to photoreceptors. We previously showed that horizontal cell density was not significantly decreased in CAST KO or CAST/ELKS double KO mice, while dendritic branching was sparse, and only a single horizontal cell tip could be seen at the ribbon synapse triad, as revealed by 3D reconstruction of electron microscopy images [8]. We assume that the reduction in contrast sensitivity in the CAST KO is caused by reduced innervation by horizontal cell tips onto the ribbon synapse [7,8]. An ectopic ribbon was observed with less dendritic branching or reduced horizontal cell density [8]. Indeed, ablation of the horizontal cell leads to photoreceptor degeneration and ectopic ribbon synapses [17].

Here, we investigated the roles of the presynaptic proteins CAST and ELKS in photoreceptor and horizontal cells. Unexpectedly, CAST and ELKS in photoreceptor cells did not play a significant role in ribbon synapse ectopic localization. Furthermore, because the horizontal cells had degenerated in the CAST KO mice, the depletion of CAST and ELKS in these cells did not affect formation of the retina. In contrast, depletion of CAST and ELKS in the mature retina caused degeneration of the photoreceptors and ectopic ribbon synapses. These findings demonstrate that photoreceptor triad synapse localization is regulated by neurotransmitter release from both photoreceptor and horizontal cells. Furthermore, they suggest that CAST and ELKS have critical roles in neurotransmitter release at photoreceptor ribbon synapses and are therefore essential for the maintenance of neural signal transduction in the retina.

## 2. Results

### 2.1. Ectopic Localization of Rod Terminals in the CAST KO Retina

**Figure 1:**
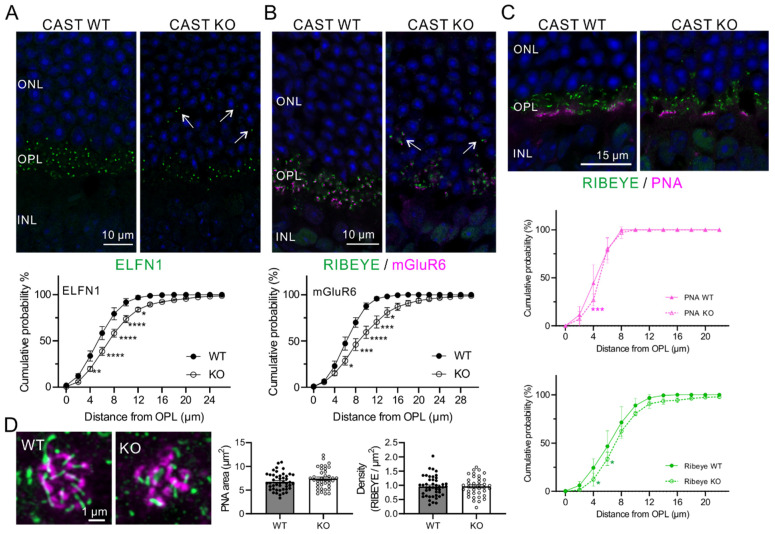
Ectopic localization of ribbon synapses in CAST KO at rod terminals. (**A**) In the WT and CAST KO retinae, the rod terminals synapsing onto ON-rod bipolar cells were labeled with anti-ELFN1 antibody. The ELFN1 signals, which were localized in the OPL in the WT, were found ectopically localized to the ONL in the CAST KO (white arrows). The signals were distributed significantly far from the bottom of the OPL in the CAST KO. Mean ± SEM; WT: *n* = 3; CAST KO: *n* = 4; * *p* < 0.05, ** *p* < 0.01, *** *p* < 0.001, **** *p* < 0.0001 (two-way ANOVA with post hoc Sidak test). (**B**) The postsynaptic sites of ON-rod bipolar cells, detected with anti-mGluR6 antibody, were ectopically localized in the ONL in CAST KO mice (white arrows). The signals were distributed significantly far from the bottom of the OPL in CAST KO. Mean ± SEM; WT: *n* = 3; CAST KO: *n* = 6; * *p* < 0.05, *** *p* < 0.001, **** *p* < 0.0001 (two-way ANOVA with post-hoc Sidak test). ONL: outer nuclear layer; OPL: outer plexiform layer; INL: inner nuclear layer. (**C**) The cone terminals labeled by PNA were mostly localized at the inner subregion of the OPL in the WT and CAST KO retinae. The distribution analysis showed that the PNA labels in the WT and CAST KO were localized to the bottom of the OPL compared with the RIBEYE labeling. Mean ± SEM; WT: *n* = 4; CAST KO: *n* = 4; * *p* < 0.05, RIBEYE (green), between WT and KO; *** *p* < 0.001, PNA (magenta), between WT and KO (two-way ANOVA with post hoc Tukey test). (**D**) The density of ribbon synapses in the cone terminals was quantified at RIBEYE signals in the PNA-labeled area. The PNA area and density of RIBEYE were not significantly different between WT and CAST KO. Mean ± SEM; WT: *n* = 45 from 4 mice; CAST KO: *n* = 38 from 4 mice.

In our previous study using CAST KO mice, we demonstrated ectopic ribbon synapses in the ONL, reduced size of ribbon synapses, and decreased Ca^2+^ signals in the photoreceptors associated with visual disturbance [8]. Here, we investigated the distribution of rod and cone terminals in the CAST KO mice. Initially, the synapses between rods and ON-rod bipolar cells (RBCs), detected by staining for the adhesion molecule ELFN1 [18,19], were ectopically distributed in the ONL, and the distance of the ELFN1 signal from the border between the INL and OPL was significantly shifted further away from the OPL in CAST KO mice (Figure 1A). Because of the key role of ELFN1 in the localization of postsynaptic mGluR6 in RBCs, the ectopic distribution of mGluR6 in the ONL was demonstrated in CAST KO mice (Figure 1B). Intriguingly, the mGluR6 labeling colocalized with RIBEYE-labeled ribbon synapses at the OPL in WT and CAST KO mice (WT = 99.7% ± 0.14, *n* = 22 from 3 mice; CAST KO = 98.3% ± 0.34, *n* = 22 from 4 mice; mean ± SEM). Notably, this colocalization was significantly reduced at ONL synapses in the CAST KO (CAST KO ONL = 94.0% ± 1.73, *n* = 21 from 4 mice; mean ± SEM; *** *p* < 0.001, vs. WT (OPL); ** *p* < 0.01, vs. KO OPL; one-way ANOVA with post-hoc Tukey’s test). Furthermore, 66.7% ± 0.14 (mean ± SEM, *n* = 31) of the ectopically-localized ribbon synapses in the ONL were colocalized with mGluR6 labeled ON-RBCs in CAST KO mice.

Next, we analyzed the cone photoreceptor terminals by labeling them with peanut agglutinin (PNA). Unlike the rod terminals, the PNA-labeled cone terminals were not distributed in the ONL (Figure 1C). Distribution analysis showed that all the PNA signal was localized in the OPL in CAST KO mice, even although the RIBEYE signal was partially localized in the ONL. The number and density of ribbon synapses of PNA-labeled cone terminals were not significantly different between WT and CAST KO animals (Figure 1D). These results suggest that CAST KO in the retina primarily affects the distribution of ribbon synapses at rod terminals.

### 2.2. Depletion of CAST and ELKS in Photoreceptor Neurons Does Not Affect Retina Formation

**Figure 2:**
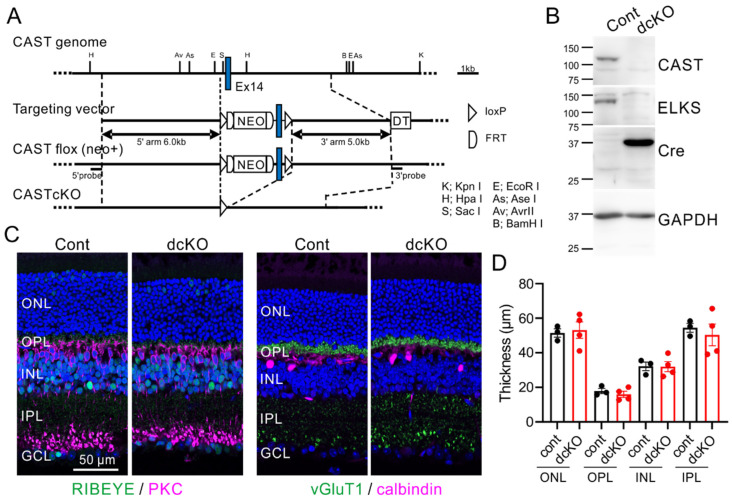
Specific depletion of CAST and ELKS from retina has limited impact on neural and synaptic distributions. (**A**) The CAST-flox allele contains two loxP sequences flanking exon 14 of mouse CAST. Conditional ablation of CAST was achieved by crossing flox mice with Cre-expressing lines. (**B**) Western blotting of adult retinal homogenates from control (CAST/ELKS double-floxed mouse (Cont)) and deletion mutant (CAST/ELKS double-floxed with Crx-Cre^+/−^ [double cKO]) mice with the indicated antibodies showed ablation of CAST and ELKS in the Cre-expressing retina. (**C**,**D**) Representative images of immunolabeled retinal sections showing photoreceptor ribbon synapses (anti-RIBEYE) and their terminals (anti-vGluT1) distributed in the OPL. Postsynaptic target neurons of the photoreceptor ribbon synapse (bipolar cells and horizontal cells) were immunolabeled with anti-PKC antibody and anti-calbindin antibody, respectively. The thickness of each layer was not significantly different between control and CAST/ELKS Crx-Cre double cKO mice. Mean ± SEM; ONL: outer nuclear layer; OPL: outer plexiform layer; INL: inner nuclear layer; IPL: inner plexiform layer; GCL: ganglion cell layer.

To assess the role of CAST and ELKS in retina formation, we analyzed double KO mice, where CAST was globally deleted and ELKS was conditionally knocked-out using a retinal Cre expressing mouse (specifically the Crx-Cre strain), as described previously [8]. The Crx-Cre mouse expresses Cre recombinase under control of a photoreceptor-specific transcription factor [20,21]. Our immunohistochemistry analysis using anti-Cre antibody revealed a weak signal in INL neurons and a strong signal in ONL photoreceptors, which facilitated the depletion of ELKS in the OPL and IPL in the ELKS flox mouse [8]. In the previous double KO mouse (CAST KO and ELKS cKO), we were concerned with unknown roles of CAST in non-neural cells, including glial cells. Here, we generated CAST-flox mice for targeted deletion of exon 14 (Figure 2A), which causes a frameshift, resulting in premature termination. Because the family of CAST and ELKS proteins are both distributed at the synaptic OPL and IPL of the retina [7,22], and have been suggested to have complementary functions [7,8,23], we crossed CAST-flox mice with ELKS-flox mice to generate CAST/ELKS-double-floxed (dflox) mice, which were then crossed with retinal Cre-expressing Crx-Cre mice. In the resulting mice with retinal double cKO in photoreceptor neurons, the CAST and ELKS proteins in the retina were eliminated, as revealed by immunoblotting (Figure 2B).

Immunohistochemistry of control (CAST/ELKS-dflox, Crx-Cre^−/−^) and double cKO (CAST/ELKS-dflox, Crx-Cre^+/−^) retinas with anti-RIBEYE and anti-vGluT1 revealed normal localization of photoreceptor ribbon synapses in the OPL (Figure 2C). Immunolabeling of postsynaptic target neurons of the photoreceptor ribbon synapse, specifically, bipolar cells (with anti-PKC antibody) and horizontal cells (with anti-calbindin antibody), did not show any significant changes, such as filopodia-like dendritic penetrations into the ONL, in the double cKO mice compared with controls (Figure 2C).The layer thickness was not significantly different between the two groups of mice (Figure 2D; Total: control = 158.5 ± 9.1, double cKO = 157.3 ± 14.6; ONL: control = 51.4 ± 2.4, double cKO = 53.1 ± 4.7; OPL: control = 17.9 ± 1.6, double cKO = 16.0 ± 1.8; *n*: control = 3, double cKO = 4; mean ± SEM), indicating that the depletion of CAST and ELKS in photoreceptor neurons does not impact the localization of triad synapses.

### 2.3. CAST and ELKS in Horizontal Cells Do Not Play a Major Role in the Formation of the Retina

**Figure 3:**
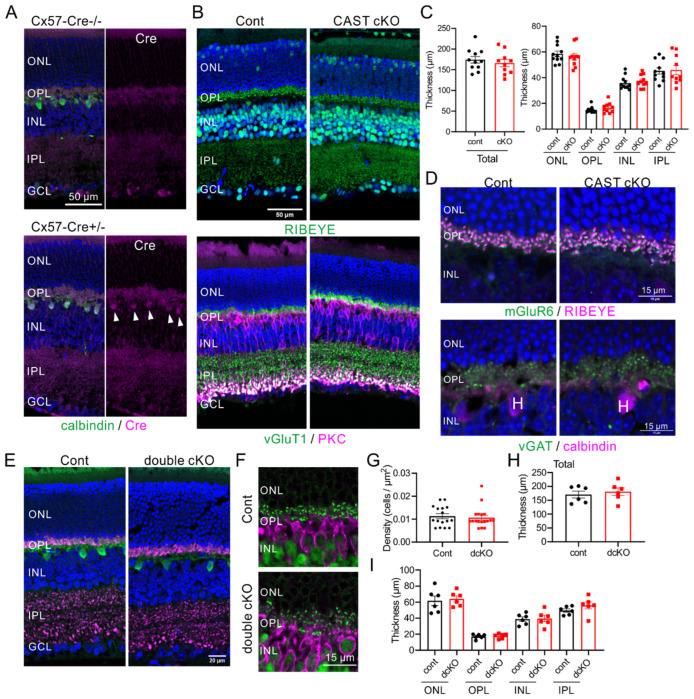
Depletion of CAST and ELKS from horizontal cells does not affect neural or synaptic distribution (**A**) In the Cx57-Cre mice, co-labeling for Cre was detected in calbindin-labeled horizontal cells in the upper part of the INL (white arrows). (**B**,**C**) Representative images of immunolabeled retinal sections showing the distribution of photoreceptor ribbon synapses (anti-RIBEYE), terminals (anti-vGluT1) in the OPL, and postsynaptic bipolar cells (anti-PKC) in the CAST-flox;Cx57-Cre^+/−^ (cKO) mice. The total thickness and thickness of each layer were not significantly different between control and cKO; mean ± SEM (Student’s *t*-test). (**D**) Immunohistochemistry showing the parallel distribution of presynaptic ribbon synapses and postsynaptic mGluR6 in the OPL. The inhibitory synapses (labeled by anti-vGAT antibody) were distributed at calbindin-labeled horizontal cell dendrites, which were unaffected in CAST cKO mice. (**E**–**I**) In the CAST-flox and ELKS-flox;Cx57-Cre^+/−^ (double cKO) mice, the horizontal cells (calbindin-positive) were localized at the upper part of the INL, and the photoreceptor terminals (anti-vGluT1) and ribbon synapses (anti-RIBEYE) were distributed in the OPL. The horizontal cell density and layer formation in the double cKO mice were not significantly different from the control; mean ± SEM (Student’s *t*-test).

In previous reports on the CAST KO mouse, we showed horizontal cell degeneration using 3D reconstruction of electron microscopy images [8]. These results indicated that the ectopic localization is caused by CAST deficiency in horizontal cells. Here, we generated a horizontal cell-specific Cre recombinase-expressing line of mice (Cx57-Cre), according to a previous report [24], and crossed these with CAST-flox mice. Immunohistochemistry using anti-Cre antibody revealed specific Cre expression in anti-calbindin positive horizontal cells in the INL (Figure 3A). Subsequently, we generated a horizontal cell CAST and ELKS double-deletion mutant (CAST/ELKS-dflox;Cx57-Cre^+/−^ [double cKO]). In retinal homogenate from this double cKO, the expression of CAST and ELKS were slightly but not significantly reduced compared with the control retina (Supplemental Figure S2). These results suggest that the majority of CAST and ELKS were expressed at the photoreceptor and bipolar cell terminal in the OPL and IPL. In the CAST-flox;Cx57-Cre^+/−^ (CAST cKO) mouse, the photoreceptor ribbon synapses (by anti-RIBEYE antibody) and terminals (by anti-vGluT1 antibody) were distributed normally in the OPL, and layer formation was not significantly different from the control (Figure 3B,C). Furthermore, the photoreceptor ribbon synapses were localized with the postsynaptic ON bipolar cell tips, as indicated by mGluR6 labeling (Figure 3D), and the inhibitory terminals, labeled by vGAT, were localized on anti-calbindin antibody labeled horizontal cell dendrites in the OPL (Figure 3D).

Next, we generated the horizontal cell CAST and ELKS double deletion mutant (CAST/ELKS-dflox;Cx57-Cre^+/−^ (double cKO)). Immunolabeling photoreceptor terminals (by vGluT1) with horizontal cells, and ribbon synapses (by RIBEYE) with bipolar cells revealed no impact of the double cKO on neuronal or synaptic distribution (Figure 3E,F). The density of horizontal cells and layer thickness did not significantly differ either (Figure 3G–I). These results suggest that CAST and ELKS deficiency in photoreceptors or horizontal cells does not directly cause the mislocalization of rod photoreceptor synapses. The ectopic synapses in the CAST KO may therefore be caused by other factors, or a combination of factors, at the photoreceptor triad.

### 2.4. Acute Ablation of CAST and ELKS Causes Photoreceptor Degeneration

**Figure 4:**
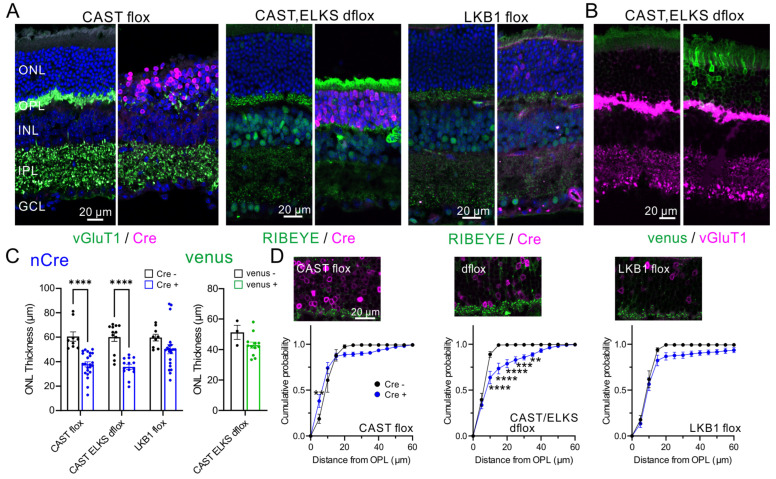
Acute depletion of CAST and ELKS causes photoreceptor degeneration in the mature retina. (**A**,**B**) Acute expression of Cre recombinase using AAV5-CAGGS-nCre in CAST flox, CAST/ELKS dflox, and LKB1 flox mouse. Subretinal injection of AAV5 produces mosaic Cre expression in the retina, which can be identified by immunolabeling for Cre (magenta). AAV-mediated Cre expression causes reduced ONL thickness, indicating photoreceptor degeneration, with ablation of CAST and ELKS. Further, RIBEYE or vGluT1 immunolabeling reveals reduced ribbon synapses in the OPL (green). As a negative control, AAV-mediated expression of Venus had no effect on layer thickness or vGluT1-labeled photoreceptor terminal in the OPL (magenta) in CAST and ELKS dflox retina. (**C**) Quantitative analysis of ONL thickness in CAST-flox, CAST/ELKS-dflox and LKB1-flox cKO mice. The thickness was significantly decreased in the CAST flox and CAST/ELKS dflox mice but was unchanged in the LKB1 flox. **** *p* < 0.0001 (two-way ANOVA with post hoc Sidak test). The AAV5-CAGGS-venus expression in CAST/ELKS-dflox mice did not affect ONL thickness (Student’s *t*-test). (**D**) Cumulative histogram of the distance probability of RIBEYE-labeled ribbon synapse distribution. In the Cre-negative region, the frequency curve reached ~100% within 15–20 μm, indicating normal ribbon synapse localization in the OPL (black line). However, in the CAST/ELKS dflox mice, the frequency curve at the Cre expressing region (presumably depletion of CAST and ELKS, blue line) was shifted to the right, with approximately 20–30% of ribbon synapses ectopically localized in the ONL. Mean ± SEM; CAST-flox: Cre negative: *n* = 5, Cre positive: *n* = 7; CAST/ELKS-dflox: Cre negative: *n* = 9, Cre positive: *n* = 8; LKB1-flox: Cre negative: *n* = 5, Cre positive: *n* = 6. ** *p* < 0.01, *** *p* < 0.001, **** *p* < 0.0001 (two-way ANOVA with post hoc Sidak test).

Next, we investigated the role of CAST in mature photoreceptors. In a previous report, acute ELKS depletion induced by subretinal injection of adeno-associated virus serotype 5 (AAV5) encoding Cre (AAV5-CAGGS-nCre) into ELKS-flox mice caused loss of ELKS at the OPL and photoreceptor degeneration [8]. These effects appear likely to be caused by the lack of ELKS and not by side effects of the virus injection, since (1) AAV-mediated Venus expression had less effect on ONL thickness; (2) Acute Cre expression in the ELKS cKO with Crx-Cre mice caused no significant change in ONL thickness; and (3) Acute Cre expression with CAST in double KO mice suppressed the reduction of ONL thickness. Here, following the method described in the previous report using the ELKS flox mice, AAV was injected into the eyes at ~5 weeks of age. The retinal morphology was then analyzed by immunohistochemistry at least 3 weeks post-injection (Figure 4). Cre expression caused a reduction in ONL thickness in the CAST or CAST/ELKS floxed mice, indicating degeneration of the photoreceptors with depletion of CAST and ELKS (Figure 4A,C). We also investigated the acute depletion of the serine/threonine kinase LKB1, which regulates the AMPK subfamily, including AMPKα, SAD-A/B, NUAK1/2, SIK1–3, MARK1–4, and SNRK. The deletion of LKB1 in the retina also caused the ectopic localization of ribbon synapses in the ONL [12]. Compared with the CAST and CAST/ELKS floxed mice, AAV-mediated Cre expression at photoreceptors in the LKB1 flox mice had less effect on ONL thickness (Figure 4A,C). As a negative control, AAV-mediated Venus expression (AAV5-CAGGS-IRES-venus) in the CAST/ELKS dflox retina had no significant effect on ONL thickness (Figure 4B,C). We found further that acute Cre expression (presumably depletion of CAST/ELKS) resulted in ectopic ribbon synapses in the ONL, which were not particularly observed in the CAST flox or LKB1 flox mice (Figure 4D).

Ectopic ribbon synapses in the ONL have been observed in a mouse model of retinitis pigmentosa, as well as in a number of mutant mouse lines, including the CAST KO and LKB1 cKO [7,8,12], suggesting an association of these aberrant structures with retinal degeneration and visual defects. Following our previous results in the ELKS flox mice, in which photoreceptor ELKS depletion caused degeneration of photoreceptors [8], here we show that the presynaptic protein CAST is involved in maintaining the photoreceptor and its synapses. Moreover, depletion of CAST and ELKS in the mature retina causes severe degeneration of the photoreceptor neural network.

## 3. Discussion

In our current study, we investigated the roles of the presynaptic proteins CAST and ELKS in the localization and degeneration of photoreceptor ribbon synapses. We showed that CAST KO mice exhibited a decrease in the size of photoreceptor ribbons, as well as ectopic localization of these ribbons in the ONL [8]. These defects likely result from disrupted Ca^2+^ channel localization, because CAST has a critical role in Ca^2+^ channel clustering at presynaptic release sites [7,8]. Notably, we showed that the ectopically-localized ribbon synapses in the CAST KO mice were primarily rod terminals, and that cone terminals were minimally affected. Furthermore, our targeted genetic ablation of CAST and ELKS revealed that, unlike the CAST KO, the depletion of these proteins in either photoreceptor or horizontal cells alone does not cause ectopic synapses. Therefore, simple disruption of CAST and ELKS in each neuronal type may be complemented by other active zone proteins, and the mechanism underlying ectopic localization of photoreceptor synapses to the ONL in the CAST KO mice requires other unknown factors.

The transsynaptic contact between presynaptic ELFN1 and postsynaptic mGluR6 plays a key role in the formation of synapses between the rod terminals and ON-RBCs. Depletion of ELFN1 completely eliminates the mGluR6 accumulation at synapses [18,19]. However, neither ELFN1 nor mGluR6 deletion in the CAST KO retina prevented the ectopic localization of rod terminals [19]. Thus, the physical apposition of ELFN1 and mGluR6 does not contribute significantly to photoreceptor remodeling in CAST KO mice.

Here, AAV-mediated genetic ablation of CAST and ELKS in the mature retina led to the degeneration of photoreceptors. The active zone is the specialized presynaptic site of neurotransmitter release. At the active zone, core proteins such as CAST and ELKS, form a large protein complex that performs multiple functions including docking and priming synaptic vesicles, recruiting Ca^2+^ channels, and tethering vesicles and other molecules to the synaptic sites. Additionally, these proteins play critical roles in synaptic plasticity [25]. Although, these core proteins have well-conserved domain structures for their specific roles, previous studies have shown that functional redundancy exists between different active zone proteins. For example, CAST/ELKS and RIM or RIM and RIM-binding protein (RIM-BP) have been found to have overlapping functions in forming the active zone [26,27]. Therefore, proteins in the presynaptic active zone have overlapping functions, and if one family of proteins (e.g., CAST and ELKS) is lost during development in genetically modified mice (Crx-Cre or Cx57-Cre mediated cKO mice), the other active zone proteins can compensate for this loss. However, there is limited research on the turnover of active zone proteins. Our study provides new insights into the metabolic processes of the presynaptic release site. Specifically, we show that acute depletion of CAST and ELKS are not compensated for by other proteins, suggesting that each active zone protein has a unique role that cannot be fulfilled in the mature ribbon synapse. Notably, when LKB1, a factor known to be involved in the formation of ectopic ribbon synapses in the photoreceptor, was eliminated in the mature retina, photoreceptor degeneration was less severe. LKB1 is a serine/threonine kinase that activates a wide variety of AMP-activated protein kinase (AMPK) pathways, and is involved in regulating neuronal polarity and axon formation [28]. During neural development, LKB1 phosphorylates and activates SAD kinase family members, including SAD-A and SAD-B, which are involved in axon formation and synaptic function [29,30]. Indeed, SAD phosphorylation is reduced in the LKB1 KO retina, and activation of AMPK signaling attenuates photoreceptor axon retraction [12]. It is worth noting that SAD-B phosphorylates CAST and ELKS to regulate synaptic vesicle release [31]. However, our initial conjecture that the formation of ectopic synapses in CAST KO mice involved the LKB1–SAD-B signaling pathway was contradicted by the lack of ectopic synapses in the photoreceptor-specific CAST/ELKS KO mice. Furthermore, the CAST/ELKS-ablation-induced degeneration of photoreceptors in the mature retina could not be reproduced by depletion of LKB1, suggesting that other pathways regulate the functions of CAST and ELKS.

The remodeling of photoreceptors caused by CAST and ELKS ablation in the mature retina sheds light on the previously unknown mechanisms of retinitis pigmentosa, a retinal disorder, with both sporadic and inherited cases, characterized by the degeneration of rod photoreceptors. Stem cell therapy has emerged as a promising approach for restoring visual function [32], including transplantation of retinal progenitor cells or photoreceptors derived from induced pluripotent stem cells. However, the invasive nature of intravitreal injection carries potential risks, and therefore, alternative therapeutic strategies to delay or prevent photoreceptor degeneration are needed. Our findings provide insight into the molecular and functional mechanisms of retinal degeneration and highlight the critical importance of these presynaptic proteins in the maintenance of neural transduction in the retina.

## 4. Materials and Methods

### 4.1. Mouse Lines

The use of animals was approved by the Institutional Committee for the Care and Use of Experimental Animals at the University of Yamanashi, and the Tokyo University of Science. The generation of CAST KO mice has been described in previous reports [7,8], and LKB1-flox mice were obtained from The Jackson Lab (Strain #014143, [33]).

CAST-flox mice were generated as follows. A genomic DNA fragment carrying exon 14 of CAST was introduced into pMC1DTpA [34]. The 1.8-kb DNA fragment carrying the 34-bp loxP sequence and phosphoglycerate kinase 1 (Pgk-1) promoter-driven neomycin phosphotransferase (neo) gene, flanked by two Flp recognition target (frt) sites, was inserted 249 bp upstream of exon 14, and the 34-bp loxP sequence was inserted 204 bp downstream of exon 14. The targeting vector contained exon 14 of the CAST gene flanked by loxP sequences, a neo gene flanked by two frts, the 6.0-kb upstream and 5.0-kb downstream genomic sequences, and 4.3-kb pMC1DTpA. The vector was linearized with SalI and electroporated into the embryonic stem (ES) cell line RENKA, derived from the C57BL/6 strain [35], as previously described [36]. G-418 (175 μg/mL)-resistant clones were selected, and recombinant clones were identified by Southern blot analysis of AseI or KpnI-digested genomic DNA using a PCR-amplified 500-bp fragment, PCR-amplified 499-bp fragment or the 0.6-kb PstI fragment from pLFNeo [37], as 5′, 3′ and *neo* probes, respectively. The CAST/ELKS-dflox mice were crossed with CAST-flox and ELKS-flox mice [38].

The inducible photoreceptor-specific deletion mutant mice were obtained by crossing these floxed mice with Crx-Cre mice carrying Cre recombinase under the control of the Crx promoter (provided by T. Furukawa, [21]). The horizontal cell-specific deletion mutant mice were crossed with Cx57-Cre mice, which were generated from Cx57-DTRfrtCre (EMMA ID; EM 06024 [17]). The frt-flanked DTR-eGFP cassette was excised in vivo by mating to Flp-deleter mice (Actb-FLPe, [39]). This brought the Cre recombinase under the direct control of the Cx57 promoter and left a single frt site in intron 1 [24]. Mice were genotyped for genes encoding CAST, ELKS flox, and Cre recombinase by PCR. Experiments were performed on adult mice (8–15 months-old), unless otherwise stated.

### 4.2. Immunohistochemistry and Image Analysis

For dissection of the retina, mice were deeply anesthetized with pentobarbital or a mixture of medetomidine hydrochloride (0.75 mg/kg; Zenoaq, Fukushima, Japan), midazolam (4.0 mg/kg; Sandoz, Tokyo, Japan) and butorphanol tartrate (5.0 mg/kg; Meiji Seika Pharma, Tokyo, Japan), and the dissected eyeballs were fixed with 4% paraformaldehyde in 0.1 M phosphate buffer (pH 7.4). The retinae were cryoprotected in 30% (*w*/*v*) sucrose in PBS for >2 h, and then pieces of retinae were sectioned at a thickness of 10 μm on a cryostat (HM525 NX, Thermo or CM3050S, Leica, Wetzlar, Germany). The collected sections on slides were blocked for 1 h in blocking solution (2% normal goat or horse serum, 10% Block Ace (Dainippon Pharmaceutical, Osaka, Japan), 0.2% Triton X-100 in PBS) and incubated in primary antibodies in blocking solution overnight at room temperature. The following primary antibodies were used: anti-RIBEYE (1:1000, rabbit, 192003, SYSY, Göttingen, Germany), anti-CtBP2 (1:200, mouse, 642044, BD Transduction, San Jose, CA, USA), anti-PKC (1:1000, mouse, ab31, Abcam, Cambridge, MA, USA), anti-vGluT1 (1:1000, guinea pig, [8]), anti-calbindin (1:2000, mouse, C9848, Sigma-Aldrich, St. Louis, MO, USA), anti-ELFN1 (1:200, rabbit, [18]), anti-Cre (1:800, mouse, MAB3120 or 1:500, rabbit, 69050-3, Millipore, Eschborn, Germany), and PNA (1:250, RL-1072, Vector Lab, Burlingame, CA, USA). The custom antibody against mGluR6 (amino acids 853–871) was generated in rabbit, and affinity-purified on an antigen-containing column (1:1000, rabbit, Scrum [40]). The specificity of the antibody was verified using mGluR6 KO retinal samples (kindly provided Dr. Furukawa, Osaka Bioscience Institute; Supplemental Figure S1). For the detection of mGluR6 and ELFN1, sections were boiled in 10 mM sodium citrate buffer (pH 6.0) for antigen retrieval and rinsed with PBS, before the primary antibody reaction. The sections were further processed with appropriate Alexa Fluor-conjugated secondary antibodies for 1 h at room temperature.

Immunolabeled samples were viewed under a confocal laser microscope (FV1200, Olympus or LSM900/Airyscan, Zeiss, Oberkochen, Germany), and analyzed using ImageJ. To measure immunolabeled synapse distribution from the OPL to ONL, fluorescence images were taken with a 60× objective and transformed into binary images. Using the ImageJ ‘Analyze Particle’ program, particle distribution was shown on the *y*-axis as the distance from the bottom of the OPL, as described previously [8]. The statistical significance between WT and CAST KO was analyzed using Student’s *t*-test. The distribution of ribbon synapses of cumulative probability was analyzed by two-way ANOVA followed by the post hoc Tukey test or Sidak test, corrected for multiple comparisons.

### 4.3. Immunoblotting

Retinal homogenates from the adult mutant mice were analyzed by Western blotting. The primary antibodies used for Western blotting were anti-ELKS (1:500, rabbit, [41]), anti-CAST (1:1000, rabbit, [42]), anti-Cre (1:500, rabbit, 69050-3, Millipore), and anti-GAPDH (1:5000, HRP conjugate, #3683, Cell Signaling Technology, Danvers, MA, USA).

### 4.4. AAV5-Mediated Cre Expression and Image Analysis

For AAV-mediated Cre expression in the retina, a plasmid encoding AAV5-CAGGS-nCre and AAV5-CAGGS-IRES-Venus was prepared as previously described [8]. To inject the virus into the eyes, mice were anesthetized at ~5 weeks of age with ketamine/xylazine by intraperitoneal injection. A 30-gauge needle was used to make a small hole in the temporal eye, below the cornea, and ~1 μL of AAV virus (8.5 × 10^9^–1.7 × 10^10^ gc/µL) was injected into the vitreous humor over the retina using a glass pipette. At least at 3 weeks post-injection, the mice were deeply anesthetized and dissected eyeballs were fixed with 4% paraformaldehyde in 0.1 M phosphate buffer (pH 7.4). Retinal slices were used for immunohistochemistry.

Because AAV-mediated Cre expression in photoreceptors was ‘patchy’ in the injected retinae, we took at least three Cre positive regions from the same retina. For ONL thickness, the images were obtained using a 40× objective lens (NA 0.95). RIBEYE density and distribution were calculated from images taken with a 60× objective and a 2× digital zoom. Data were compared between Cre-positive and Cre-negative regions using two-way ANOVA or Student’s *t*-test, as appropriate.

## Data Availability

The datasets generated during the current study are available from the corresponding authors on request.

## Supplementary Materials

The supporting information can be downloaded at: https://www.mdpi.com/article/10.3390/ijms24087251/s1.
