# Supplementary material for: Critical Role of the Presynaptic Protein CAST in Maintaining the Photoreceptor Ribbon Synapse Triad
Source: Int J Mol Sci. 2023 Apr 14;24(8):7251. doi: 10.3390/ijms24087251 (PMC10138387; doi:10.3390/ijms24087251)
Supplement: Supplementary file 1 [file ijms-24-07251-s001.zip › ijms-2293613-supplementary.pdf]

Communication

Critical role of the presynaptic protein CAST in maintaining the photoreceptor ribbon synapse triad

Akari Hagiwara[1, 2, *], Ayako Mizutani[1], Saki Kawamura[1], Manabu Abe[3], Yamato Hida[2], Kenji Sakimura[3] and Toshihisa Ohtsuka[2, *]

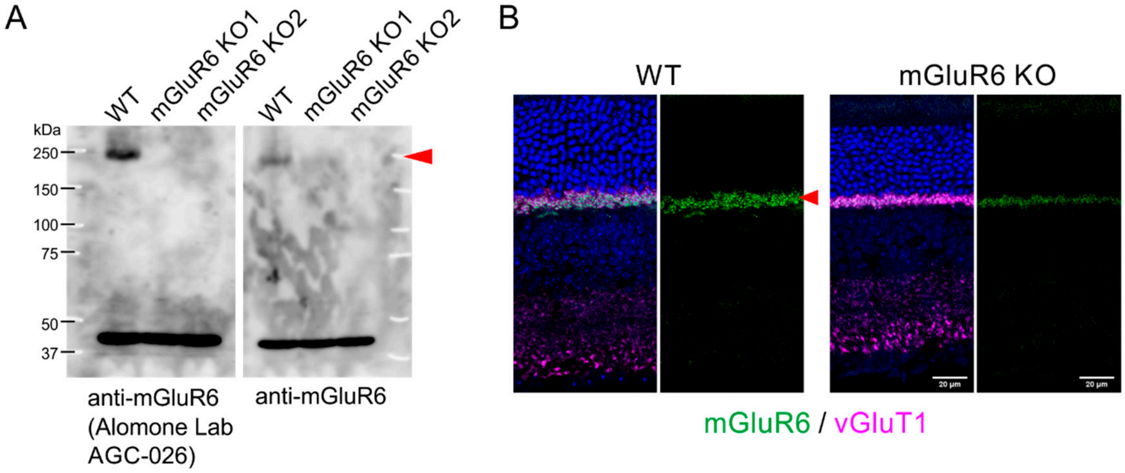

Figure S1. Specificity of mGluR6 antibody (A) Western blotting of retinal homogenates from control and mGluR6 KO mice (KO1, KO2) confirmed the specificity of the anti-mGluR6 antibody from Alomone Labs (AGC-026) and our custom-made antibody (arrowhead). **(B)** For immunostaining, the specificity of the anti-mGluR6 antibody was confirmed on retinal sections. The bright punctate signals in the OPL were colocalized with vGluT1 in the control retina and were absent in the mGluR6 KO mouse. The Western blotting homogenate and fixed retinae from mGluR6 KO mice were kindly provided by Professor T. Furukawa.

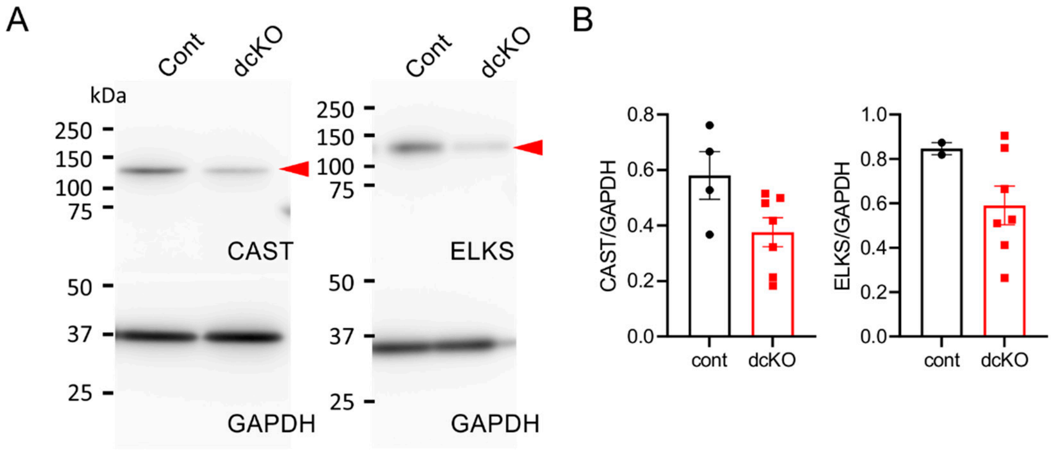

Figure S2. Depletion of CAST and ELKS in the retina of CAST/ELKS dflox mice crossed with Cx57-Cre mice. (A) Western blotting of retinal homogenates from CAST/ELKS dflox (cont) and CAST/ELKS dflox;Cx57-Cre$^{+/-}$ (dcKO) mice using the indicated antibodies. **(B)** Band intensities of CAST and ELKS were normalized with GAPDH and compared between control and dcKO. Expression was slightly but not significantly reduced in the dcKO mice.
